# Molecular basis of mucopolysaccharidosis type II (Hunter syndrome): first review and classification of published *IDS* gene variants

**DOI:** 10.1186/s40246-024-00701-w

**Published:** 2024-12-02

**Authors:** Alessandra Zanetti, Francesca D’Avanzo, Rosella Tomanin

**Affiliations:** 1 Laboratory of Diagnosis and Therapy of Lysosomal Disorders, Department of Women’s and Children’s Health, University of Padova, Padova, Italy; 2 Fondazione Istituto di Ricerca Pediatrica Città della Speranza, Padova, Italy

**Keywords:** Mucopolysaccharidosis type II, MPS II, Hunter syndrome, Iduronate 2-sulfatase, *IDS*, *IDS* variants, *IDSP1*, Recombination, Lysosomal storage disorder, Variants classification, Enzyme replacement therapy

## Abstract

**Purpose:**

Mucopolysaccharidosis type II (MPS II) is a rare X-linked lysosomal storage disorder caused by genetic alterations in the iduronate 2-sulfatase (*IDS*) gene. A wide range of variants has been reported for different countries and ethnic groups. We collected, analyzed and uniformly summarized all published *IDS* gene variants reported in literature up to June 2023, here providing the first worldwide review and classification.

**Methods:**

Data was obtained from a literature search, conducted in PubMed and Google. All data was analyzed to define the most common alleles, geographic distribution and genotype-phenotype correlation. Moreover, point variants were classified according to their pathogenicity, based on the ACMG guidelines.

**Results:**

Several types of variants have been described in the *IDS* gene, including intrachromosomal homologous recombination occurring between the homologous regions of *IDS* gene and its pseudogene *IDSP1.* Overall, we collected 2852 individuals from 2798 families, including 24 female patients. Most families carried missense variants, followed by large deletions-insertions and complex rearrangements, small frameshift deletions/insertions and nonsense variants. Based on ACMG guidelines, 62.9% of the 779 point variants were classified as “pathogenic”, 35.4% as “likely pathogenic”, and the remaining 13 variants as having “uncertain significance”.

**Conclusion:**

Data from this study confirmed that MPS II is a genetically very heterogeneous disorder, making genotype-phenotype correlation very challenging and in most cases merely unfeasible. Mutation updates are essential for the correct molecular diagnosis, genetic counseling, prenatal and preimplantation diagnosis, and disease management.

**Supplementary Information:**

The online version contains supplementary material available at 10.1186/s40246-024-00701-w.

## Introduction

Mucopolysaccharidosis type II (MPS II, MIM # 309900), or Hunter syndrome, is a rare, life threatening neurometabolic disorder, mainly affecting children. The disease is due to pathogenic variants of the gene iduronate 2-sulfatase (*IDS*), causing in turn the deficit of the corresponding lysosomal hydrolase, required to degrade the glycosaminoglycans (GAGs) heparan- and dermatan-sulfate [1]. The lack of the functional enzyme causes a pathological accumulation of the two undegraded, or partially degraded, GAGs within the cell lysosomes and in the extracellular matrix. This represents the most evident phenomenon observed in the diseased tissues and organs, although in recent years it has become evident that other molecular mechanisms are possibly involved in the onset and progression of the disease [2].

MPS II belongs to the group of the Lysosomal Storage Disorders (LSDs), and it is inherited as an X-linked trait, thus mostly affecting male subjects, although a few cases of female patients have been described [3, 4]. Its diffusion may vary in the different areas of the world, with an incidence ranging from 0.38 per 100,000 live newborns in Brazil to 1,09 in Portugal; in general, the incidence being much lower in the European with respect to East-Asian countries [3].

Clinically, the IDS deficit causes multisystem manifestations, determining a quite heavy phenotype, which, although presenting a continuum of severity, is commonly classified into attenuated and severe forms, the latter representing about two-thirds of all patients [5, 6]. Organomegaly, cardio-respiratory impairment, coarse facial features, skeletal deformities and joint stiffness, growth retardation with short stature represent the most common traits. In addition, a heavy neurological impairment characterizes the severe forms, with progressive cognitive disabilities and behavioral problems [7]. The disease commonly shows up around 2 to 4 years of age and, in the absence of any possible treatments, it may lead to death within the second decade of life, especially in the severe forms. Attenuated forms usually show a slower progression of systemic signs and symptoms, and no or a few signs of neurological involvement, as deafness and retinal degeneration; some patients may survive into the fifth or sixth decade of life, although death usually occurs early in adulthood [8].

Together with symptomatic therapies, starting 2006 a protocol of enzyme replacement therapy (ERT) has been approved by FDA and it still remains the most applied and successful [9]. Unfortunately, its most important limitation is represented by the inability to treat the CNS impairment affecting the severe forms, since the enzyme cannot cross the blood-brain barrier (BBB) [10]. More recently, a modified formulation of recombinant IDS, where the enzyme is fused to antibodies binding BBB receptors, thus allowing the enzyme transcytosis, has been proposed. At the moment only Pabinafusp alfa, where IDS is fused with an anti-human transferrin receptor antibody, has completed the Phase III clinical trial and has obtained the authorization for use in the patients [11]. Hematopoietic stem cell transplantation, which, instead, has the ability to reach the brain and is successfully applied to treat mucopolysaccharidosis type I (Hurler syndrome), did not show much efficacy in MPS II and therefore it is rarely applied. Nevertheless, it remains a therapeutic option for the disease in some countries, as in China [12], Japan [13] and Brazil [14], and more rarely in the USA [10].

Other protocols were proposed and evaluated along the years, including a gene therapy approach, genome editing and others [15].

Administration of therapeutic approaches showed the best results when applied precociously, which needs a correct early diagnosis, quite rare for MPS II. Therefore, it often happens that therapy is applied when some irreversible damage is already established in the tissues, this reducing the possible therapeutic effect [3].

Diagnostic process for the disease is not usually straightforward since it requires the recognition of signs and symptoms, sometimes not specific for the disease since they overlap with those of other similar diseases. In addition, the rarity of MPS II does not help easy suspects, since symptoms may not recall the specific syndrome among the many neurometabolic disorders. In any case, diagnosis commonly starts from a clinical suspect, followed by biochemical tests evaluating urinary GAG level, and subsequently by enzymatic tests. Once the suspect is biochemically confirmed, molecular testing completes the diagnosis [16].

### *IDS* gene

The *IDS* gene (HGNC ID:5389; ENSG00000010404) spans 44 kb, it is structured in nine exons and is located in the chromosomal region Xq28 [17–19].

It encodes for the IDS enzyme (EC:3.1.6.13) which catalyses the hydrolysis of the C2-sulphate ester bond of 2-O-sulfo-α-L-iduronic acid residues in dermatan-sulfate and heparan-sulfate [20]. The enzyme is a 550 amino acid polypeptide, which is then processed into a 517 amino acid mature protein [20]. The crystal structure of the clinical-grade recombinant enzyme is a monomeric 76 kDa glycosylated protein, composed by two subdomains. The N-terminal subdomain SD1 (amino acid 34–443, 42 kDa ‘heavy’ chain) [21], contains the catalytic core and it is stably associated with the subdomain SD2 (residues 455–550; 14 kDa ‘light’ chain), forming a large hydrophobic packing interface [20]. The key catalytic residue of the active site is cysteine 84 which is post-translationally modified to formyl-glycine [20].

A pseudogene called *IDSP1* is located 3.9 kb from *IDS* on the telomeric side and in the opposite orientation, and it contains sequences homologous to exons 2 and 3, and to introns 2, 3, and 7, with exon 3 showing 100% sequence identity with the gene [22, 23]. The high level of similarity between the homologous regions of *IDS* and *IDSP1* is known to cause genetic rearrangements due to intrachromosomal homologous recombination events, thus complicating genetic molecular testing [24]. Moreover, *IDS* and *IDSP1* map at Xq28, a chromosomal region prone to rearrangements, given the presence of several long, homologous sequence-repeated regions [25].

Including all variant types, 792 variants have been reported up to date in the *IDS* gene by HGMD professional 2022.3, most of which being missense variants.

As previously done by our group for the *ARSB* and *GALNS* genes, whose variants cause MPS VI and MPS IVA, respectively [26, 27], in this study we uniformly collected and summarized all published *IDS* gene variants. Collected data were analyzed to determine the most common variants, their geographic distribution, and, when possible, to establish a genotype-phenotype correlation. Moreover, variants were classified according to the American College of Medical Genetics and Genomics and the Association for Molecular Pathology (ACMG/AMP) criteria [28], with some modifications, to define their pathogenicity and, finally, they were submitted to ClinVar. Overall, we collected from the literature and analyzed the genotypes of 2852 subjects affected by MPS II, from 2798 families, revealing a complex of 779 unique point variants.

At the best of our knowledge, this is the first study reporting such a comprehensive worldwide analysis. A summary and classification of all *IDS* variants would likely help in the interpretation of molecular genetics results, thus aiding to confirm the diagnosis of MPS II in suspected patients as well as helping genetic counseling in families at risk.

## Methods

### Literature search

A literature search was performed in PubMed and Google using the search terms ‘*IDS* variants’, ‘*IDS* mutations’ and similar. The obtained results were limited to studies in humans that reported subjects clinically suspected to be affected by MPS II or biochemically and/or genetically diagnosed with Hunter syndrome, according to the knowledge and/or the clinical guidelines available at the time of the publication. In addition, neonates that tested positive by newborn screening (NBS) and whose diagnosis was further confirmed by a second-tier test were collected. Last search was performed on June 2023. Each publication meeting the search criteria was screened by two independent reviewers for information regarding *IDS* variants. Reports of subjects with *IDS* variants, where variants were reported as linked to MPS II, were extracted and assessed to reduce redundancy where possible. In case of papers reporting data or part of data in an aggregated form, all possible efforts were made to report them in a disaggregated form.

### Variants annotation and correction of misreported variants

Variants were annotated according to the guidelines of the Human Genome Variation Society (HGVS) nomenclature, Version 20.05 [29] using the GRCh38.p14 genome assembly and the NC_000023.11 chromosome X sequence. For the description of sequence variants, we used reference sequence NM_000202.8 [30] for the *IDS* gene and the corresponding protein sequence NP_000193.1 (P22304) [31]. Nucleotide numbering reflects cDNA numbering with position + 1 corresponding to the A of the ATG translation initiation codon at nucleotide 170. Variant annotations were validated by Name Checker [32] for exonic variants, and by Variant Validator [33] for intronic variants. Variants reported according to the old nucleotide annotation were converted to the new one, while variants reported only at the amino acid level were annotated at nucleotide level using the experimental tool Back Translator [34]. All misreported variants and other discrepancies were corrected accordingly, when possible.

Since for most large deletions/insertions and rearrangements (mainly recombinations between *IDS* and *IDSP1* genes) no boundaries were reported at nucleotide levels, it was not possible to figure out the unicity of each genetic alteration; thus, for the sake of simplicity, they were clustered in subgroups according to pattern similarity inferred by each article.

### Variants’ classification according to ACMG/AMP guidelines and ClinVar submission

*IDS* variants clinical classification was performed according to ACMG/AMP recommendations [28]. In addition, the recommendations of the Sequence Variant Interpretation Working Group of the ClinGen initiative were followed [35], taking into consideration the specifications developed by the Lysosomal Diseases Variant Curation Expert Panel for Pompe Disease and *GAA* gene (version 2). These guidelines were here used as starting point to develop the specifications of the ACMG/AMP criteria for the *IDS* gene and Hunter syndrome, used for the present classification and reported in Supplementary File - Table S1. Additional supporting information on the variants was collected from the literature and from public databases (gnomAD, dbSNPs, ClinVar, UniProt, etc.). Supporting evidence included enzyme activity and/or results of in vitro functional studies, patients’ ethnicity, allele frequency and enzyme structure. All variants were then submitted to ClinVar database [36] with associated evidence and literature references to make them publicly available (ClinVar accession numbers: from SCV005088913 to SCV005089669 and from SCV000929879.1 to SCV000929889.1).

## Results and discussion

### *IDS* variant spectrum

**Fig. 1:**
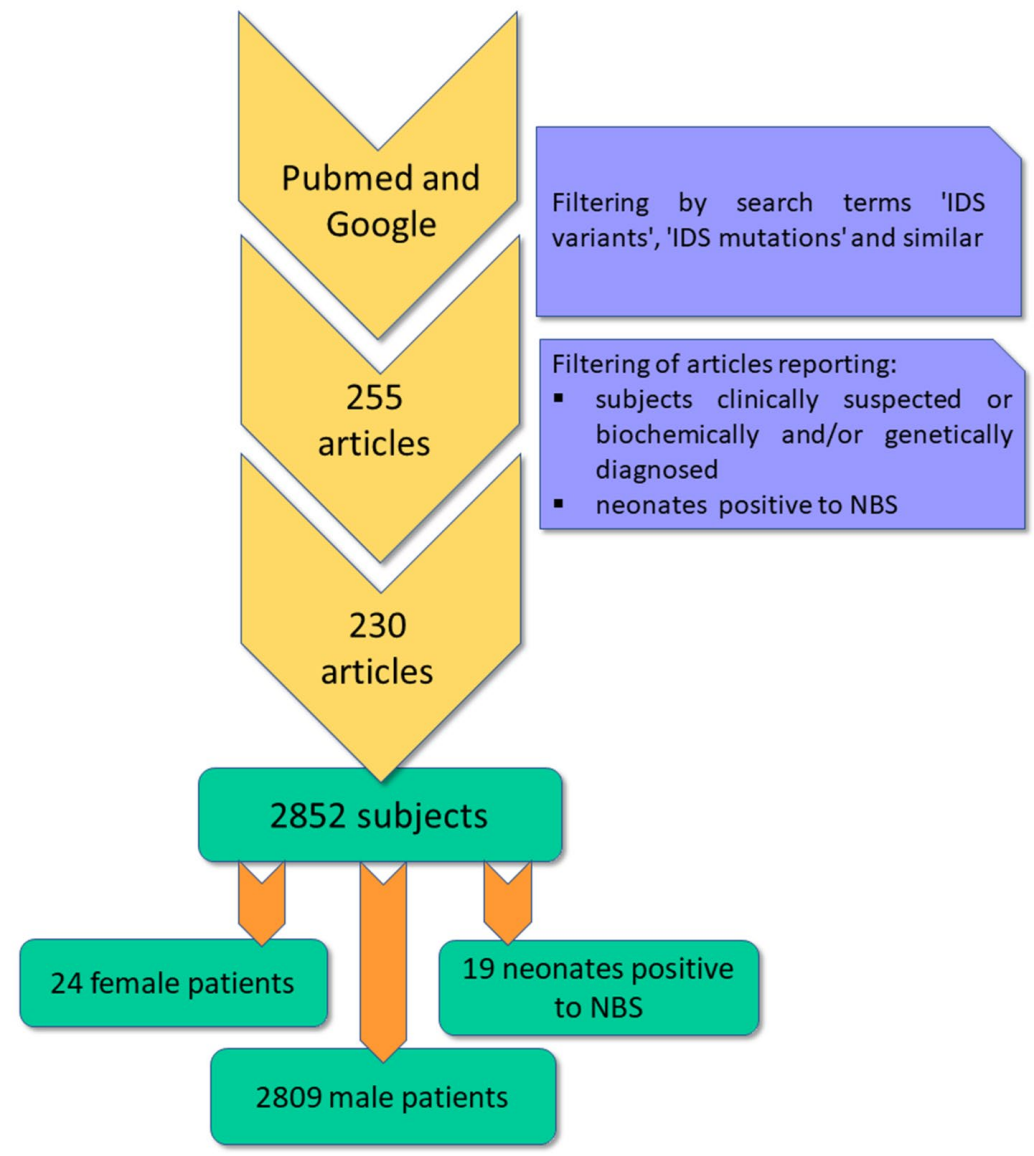
Flowchart reporting the collection and filtering of the articles analyzed as well as the number of subjects included in the study

A Pubmed and Google literature search was performed and, overall, 255 articles were collected; among these, 230 articles meeting our criteria were in depth evaluated (Fig. 1). Overall, 2852 individuals suspected or diagnosed with Hunter syndrome were collected; among them 24 female Hunter patients were described (Supplementary File - Table S2). In addition, 19 neonates that tested positive by NBS and whose diagnosis was further confirmed by second-tier tests were collected. Among the whole number of patients, 80 related subjects belonging to 29 families were reported; more specifically, 19 couples of siblings, 4 groups of 3 siblings, 1 family with 16 members, 1 family with 4 members, 2 families with 3 members and 2 families with 2 members were reported. Thus, the total number of families evaluated is 2798.

A number of 2133 families carried point variants, while 424 carried large deletions/duplications or complex rearrangements. For the remaining 241 subjects/families evaluated, either no variants were detected or reported, or the genetic analyses were not carried out. In addition, patients with a normal Southern blot pattern for whom no further analyses were described, were included in this group. 779 unique point variants were detected, while for large deletions/duplications or complex rearrangements, it was not feasible to define the unicity of all genetic alterations as, for most of them, the boundaries were not defined at nucleotide level. Therefore, these alterations were grouped per pattern similarity, as described in the related section, below.

Once collected, the *IDS* variants reported in the articles were checked for their annotation. Annotation check of point variants was performed by Name Checker and Variant Validator, revealing several misreported variants that, when possible, were corrected accordingly. However, for some variants it was not possible to obtain a consistent annotation, given the erroneous or incomplete annotation reported in the original article, mainly due to the lack of guidelines for the correct annotation at the time of publication. In several cases, variants reported in a textual form were interpreted and properly annotated.

**Fig. 2:**
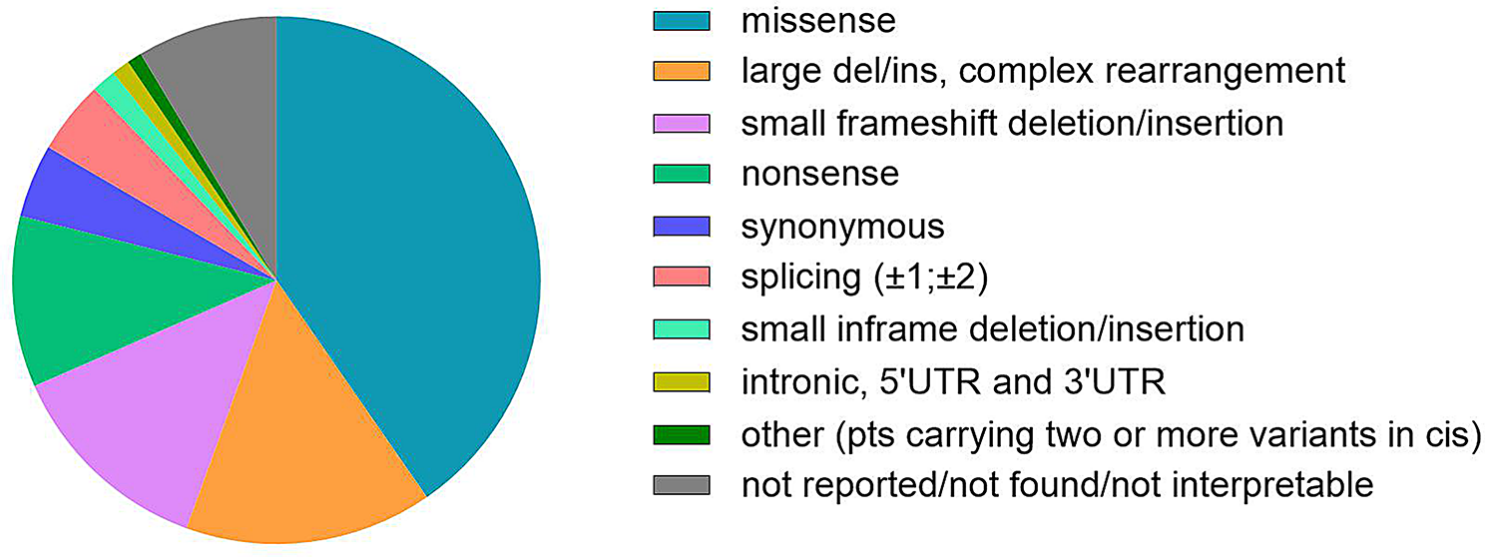
Distribution of families (n = 2798) per variant type

The analysis of the distribution by variant type of the 2798 families is reported in Fig. 2.

Most families (1129; 40.4%) carried missense variants, followed by large deletions-insertions and complex rearrangements (424 pts; 15.2%), small frameshift deletions/insertions (360 pts; 12.9%), nonsense (295; 10.5%), synonymous (126 pts, 4.5%), splicing (125 pts; 4.5%), small inframe deletions/insertions (42 pts; 1.5%), intronic, 5’ and 3’ UTR-located variants (31 pts; 1.1%), startloss (1 pt; 0.04%). In addition, 24 patients (0.9%) carried two or more variants in cis. Figure 2 also includes 241 families (8.6%), where variants were not reported, not found or not recoverable from the text of the publication, being some of these papers mainly clinical works, mostly reporting phenotypic features of the patients. In addition, more than half of these cases were described before 1994, when molecular diagnosis of the patients was mainly conducted by Southern blot analysis. Furthermore, in about one hundred cases, though published in the following years, variants were not found or, if found, they were not reported, since they were not novel [37] and therefore likely considered of no interests for the readers. Finally, in a few cases only the polymorphism c.438 C > T [p.(Thr146Thr)] was identified/reported, but no pathogenic variants were described.

The 19 neonates detected by NBS and confirmed positive by a second-tier test, showed a spectrum of 10 variants, one of which is an *IDS-IDSP1* recombination associated with inversion [38]. As for the other 9, four of them had been previously described in Hunter patients (c.817 C > T, c.1025 A > G, c.311 A > T and c.1400 C > T). The other 5 variants were only identified in NBS cases (c.142 C > T, c.1405 C > G, c.779 C > G, c.254 C > T and c.1007–1666_c.1180 + 2113delinsTT). An extremely low enzyme activity was referred for all of these 9 subjects, although some of them were still asymptomatic at the time of the second evaluation [39]. Instead, for three of them (carrying the variants c.142 C > T, c.1405 C > G and c.779 C > G) the clinical confirmation was already obtained at the time of first description, with a diagnosis of a mild form [40]. The detection of absent or extremely low IDS enzyme activity in NBS, confirmed by a second-tier test, should provide enough certainty on the Hunter diagnosis, considering that only a few pseudodeficit cases have been described so far for the disease [39].

### *IDS* point variants

**Fig. 3:**
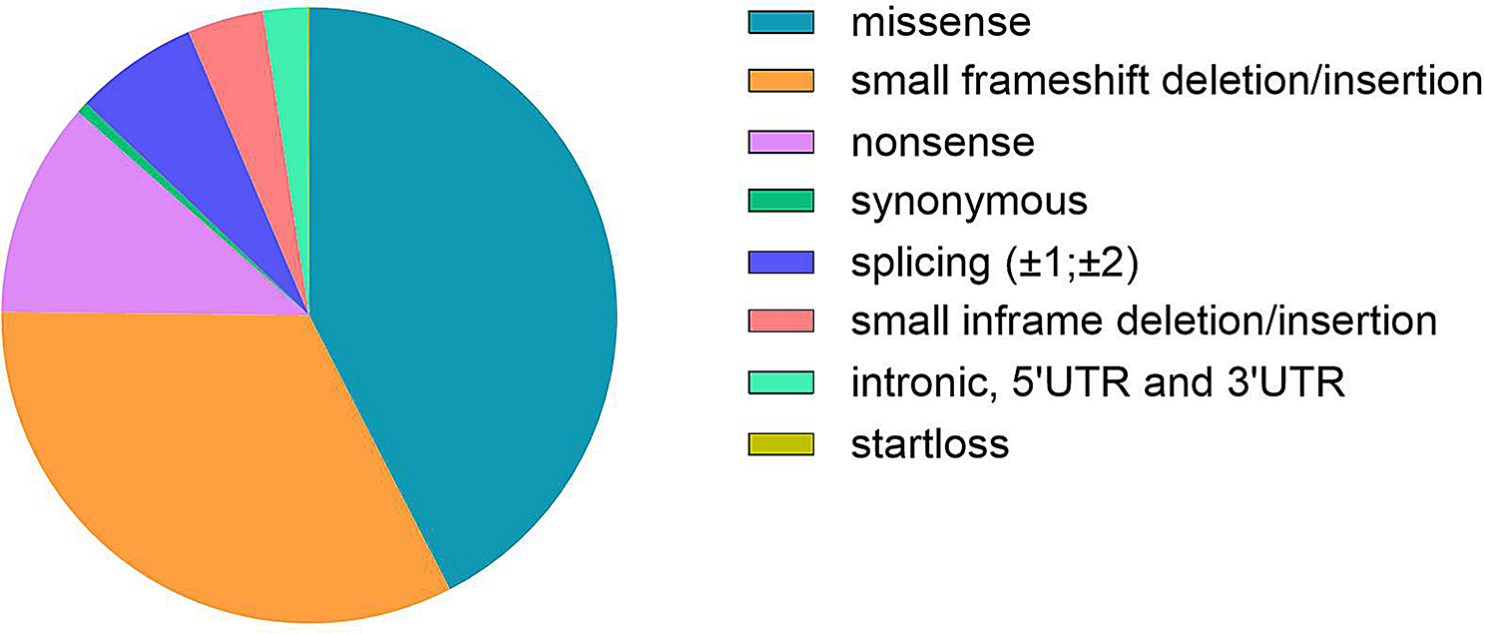
Distribution of *IDS* gene unique point variants (n = 779) per variant type

On the whole, we collected 779 unique point variants (Supplementary File - Table S3), distributed as reported in Fig. 3: 42.4% missense, 32.7% small frameshift deletion/insertions, 11.3% nonsense, 6.5% splicing, 4.0% small inframe deletions/insertions, 2.3% intronic or involving 5’UTR or 3’UTR, 0.6% synonymous, 0.1% startloss.

**Fig. 4:**
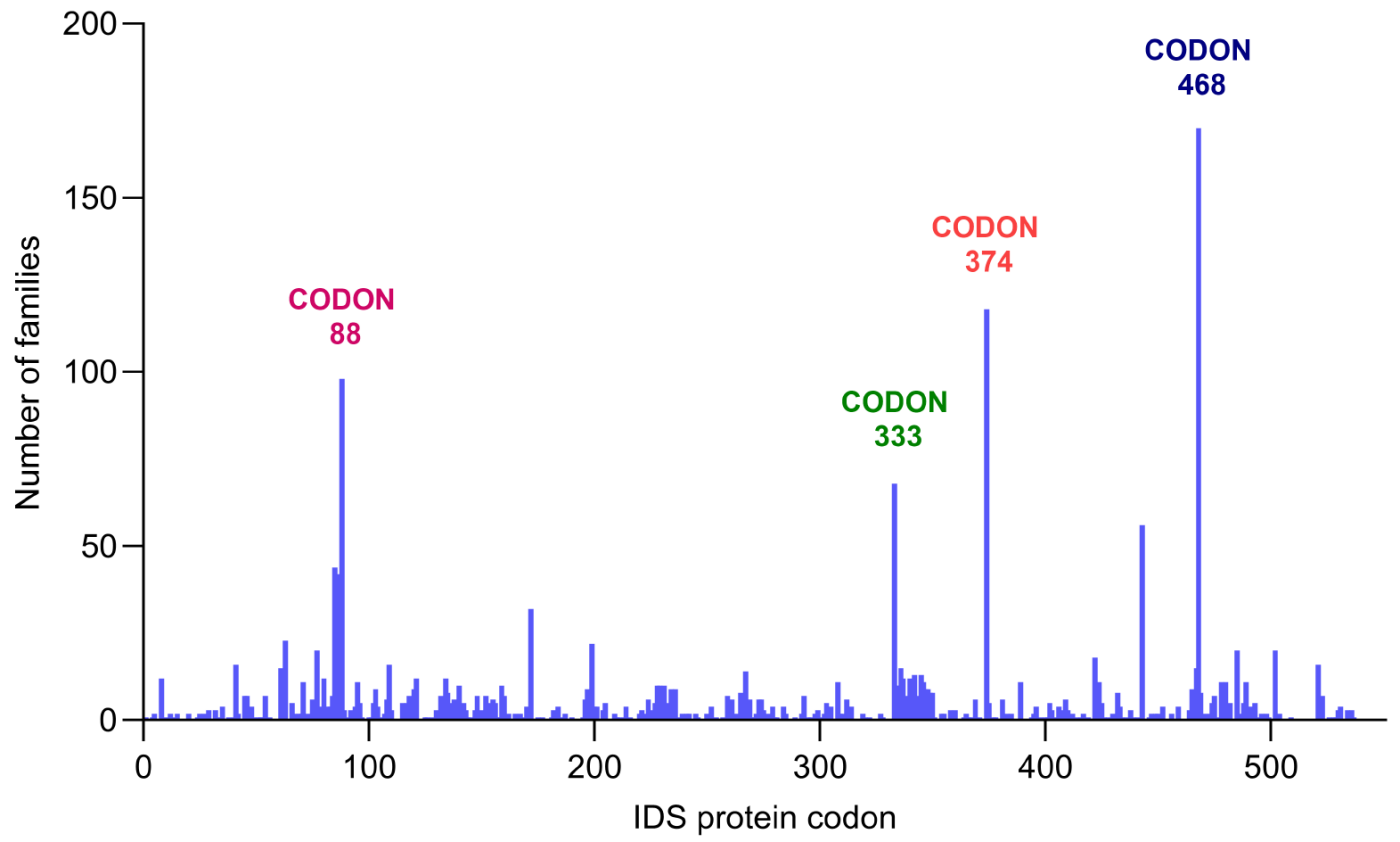
Schematic representation of the distribution of exonic point variants on the IDS protein

As depicted in Fig. 4, although *IDS* exonic point variants occurred throughout the length of the *IDS* gene, several hotspot codons are apparent: codon 468 (6 different variants, overall frequency *n* = 170 families), codon 374 (1 variant, *n* = 120 families), codon 88 (6 variants, *n* = 90 families) and codon 333 (4 variants, *n* = 68 families) (Table 1). Moreover, if we consider single exons, the more affected is exon 7 with 0.35 point variants per basepair, followed by exons 3 and 4 (0.31 and 0.21 variants/bp respectively); the least affected is exon 6 with 0.04 variants/bp.

**Table. 1:** *IDS* point variants located in the hotspot codons

Codon	88	333	374	468
Variant 1	c.262 C > T [p.(Arg88Cys)]	c.998 C > T [p.(Ser333Leu)	c.1122 C > T [p.Glu375_Gly394del]	c.1403G > A [p.(Arg468Gln)]
Variant 2	c.263G > A [p.(Arg88His)]	c.998 C > A [p.(Ser333*)]	-	c.1402 C > T [p.(Arg468Trp)]
Variant 3	c.263G > C [p.(Arg88Pro)]	c.998 C > G [p.(Ser333Trp)]	-	c.1403G > T [p.(Arg468Leu)]
Variant 4	c.262 C > G [p.(Arg88Gly)]	c.996del [p.(Ser333Argfs*7)]	-	c.1402 C > G [p.(Arg468Gly)]
Variant 5	c.263G > T [p.(Arg88Leu)]	-	-	c.1403G > C [p.(Arg468Pro)]
Variant 6	c.262 C > A [p.(Arg88Ser)]	-	-	c.1402del [p.(Arg468Glyfs*15)]

**Fig. 5:**
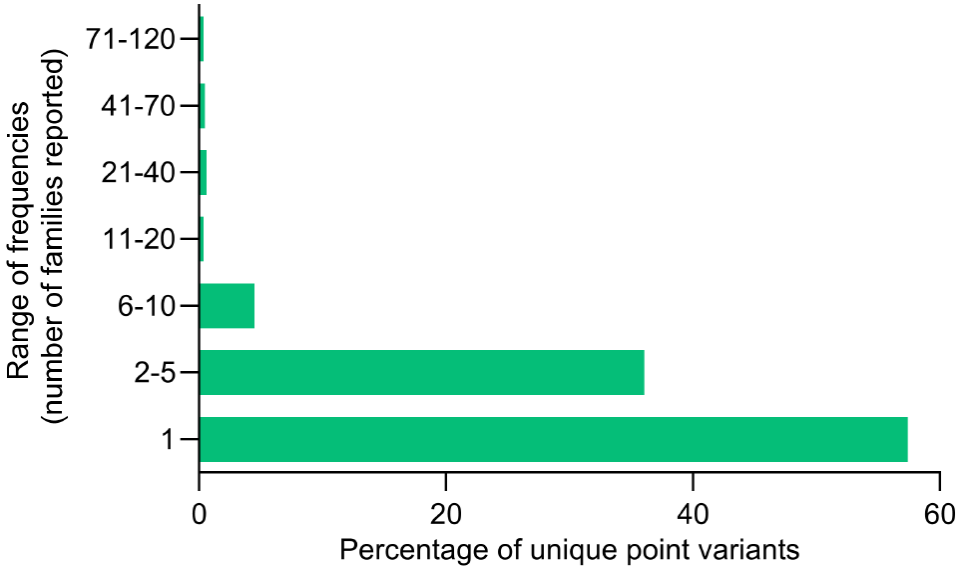
Distribution of *IDS* gene unique point variants per range of frequencies

As regarding the distribution of all unique point variants per range of frequencies, more than one half (57.4%) were reported only in one family and 36.1% were detected in a range of frequency going from 2 to 5 families. Only the remaining 6.5% unique point variants were reported with a frequency ranging from 6 to 118 families (Fig. 5).

As shown in Table 2, the ten most reported point variants were seven missense, two nonsense and one synonymous and, overall, they were detected in 20.9% of all families. The most frequent point variant was the synonymous variant c.1122 C > T [p.Glu375_Gly394del] which was described in 118 families worldwide, followed by c.1403G > A [p.(Arg468Gln)] detected in 87 families and by c.1402 C > T [p.(Arg468Trp)] reported in 73 families. c.998 C > T [p.(Ser333Leu] and c.1327 C > T [p.(Arg443*)] were reported in 64 and 54 families respectively.

**Table 2:** First ten most frequently described point variants by reported ethnicity/country

Alleles by country/ethnicity	Number detected	Percentage of that allele’s total	Percentage of all detected alleles
**c.1122 C > T [p.Glu375_Gly394del]**	**118**	**100**	**4,2**
Russian	16	13,6	0,6
Brazilian	14	11,9	0,5
Chinese	13	11,0	0,5
Indian	10	8,5	0,4
Korean	8	6,7	0,3
Spanish	7	5,8	0,2
Argentinian	5	4,2	0,2
Portuguese	4	3,4	0,1
Israelian	3	2,5	0,1
Italian	3	2,5	0,1
Japanese	3	2,5	0,1
*all other countries*	6	5,1	0,2
*not reported*	26	22,0	0.9
**c.1403G > A [p.(Arg468Gln)]**	**87**	**100**	**3**,**1**
Chinese	14	16,1	0,5
Russian	11	12,6	0,4
Argentinian	8	9,2	0,3
Italian	7	8,0	0,2
Japanese	6	6,9	0,2
Brazilian	5	5,7	0,2
Taiwanese	4	4,6	0,1
Filipino	3	3,4	0,1
Indian	3	3,4	0,1
Mexican	3	3,4	0,1
UK	3	3,4	0,1
USA	3	3,4	0,1
*all other countries*	11	12,6	0,4
*not reported*	6	6,9	0,2
**c.1402 C > T [p.(Arg468Trp)]**	**73**	**100**,**0**	**2**,**6**
Chinese	17	23,3	0,6
Brazilian	13	17,8	0,5
Indian	6	8,2	0,2
Russian	5	6,8	0,2
Taiwanese	4	5,5	0,1
*all other countries*	10	13,7	0,4
*not reported*	18	24,7	0,6
**c.998 C > T [p.(Ser333Leu)]**	**64**	**100**,**0**	**2**,**3**
Chinese	12	18,8	0,4
Brazilian	11	17,2	0,4
Russian	8	12,5	0,3
Indian	6	9,4	0,2
*all other countries*	19	29,7	0,7
*not reported*	8	12,5	0,3
c.1327 C > T [p.(Arg443*)]	**54**	**100**,**0**	**1**,**9**
Chinese	11	20,4	0,4
Brazilian	10	18,5	0,4
Indian	4	7,4	0,1
Korean	4	7,4	0,1
Russian	4	7,4	0,1
*all other countries*	11	20,4	0,4
*not reported*	10	18,5	0,4
**c.262 C > T [p.Arg88Cys]**	**44**	**100**,**0**	**1**,**6**
Chinese	10	22,7	0,4
Brazilian	6	13,6	0,2
Russian	6	13,6	0,2
*all other countries*	12	27,3	0,4
*not reported*	10	22,7	0,4
**c.263G > A [p.(Arg88His)]**	**42**	**100**,**0**	**1**,**5**
Indian	11	26,2	0,4
Russian	9	21,4	0,3
Chinese	5	11,9	0,2
Brazilian	4	9,5	0,1
Italian	3	7,1	0,1
*all other countries*	7	16,7	0,2
*not reported*	3	7,1	0,1
**c.253G > A [p.(Ala85Thr)]**	**37**	**100**,**0**	**1**,**3**
Brazilian	5	13,5	0,2
Chinese	5	13,5	0,2
Indian	5	13,5	0,2
Russian	5	13,5	0,2
Japanese	4	10,8	0,1
*all other countries*	10	27,0	0,4
*not reported*	3	8,1	0,1
**c.257 C > T [p.(Pro86Leu)]**	**31**	**100**,**0**	**1** **1**
Chinese	6	19,4	0,2
Brazilian	4	12,9	0,1
Russian	4	12,9	0,1
*all other countries*	15	48,4	0,5
*not reported*	2	6,5	0,1
c.514 C > T [p.(Arg172*)]	**31**	**100**,**0**	**1** **1**
Chinese	8	25,8	0,3
Russian	8	25,8	0,3
Indian	3	9,7	0,1
*all other countries*	6	19,4	0,2
*not reported*	6	19,4	0,2

First ten most frequent point variants by reported ethnicity/country. For each variant, only countries/ethnicities with ≥ 3 detected alleles are listed. The other countries/ethnicities are included in the category “*all other countries”*

### Large deletions/duplications and complex rearrangements involving *IDS* gene

Overall, we collected 187 patients carrying large deletions/duplications, and 238 patients carrying complex rearrangements, for a total of 425 cases, the 14.9% of all described patients. However, analysis of these two classes of variants could not be conducted in detail, as it was previously described for point variants, since for most of them the nucleotide edges were not precisely described and, in many cases, not even investigated. In addition, in the last 30 years the annotation used to describe the variants has widely changed, and this may presently result in a difficult or limited comprehension of the variants described in the original papers.

As for large deletions/duplications, they were more frequently located in exons 6, 4 and 5 respectively, although this class of genetic alterations affects all exons, less the promoter region. Due to the undefined boundaries reported in most cases, their unicity could not be unequivocally determined.

Also, for complex rearrangements, it was not feasible to define the unicity as, for most of them the boundaries of the rearranged sequences were not defined at nucleotide level. Thus, for the sake of simplicity, we grouped complex rearrangements in 6 subgroups according to the pattern similarity that was possible to infer by the article reporting each variant (Table 3**).** The first 4 groups include different types of recombinational events likely caused by the great level of similarity of *IDS* gene and its pseudogene *IDSP1*: on the whole, we collected 180 patients carrying this type of rearrangements. This represents a sort of peculiarity of this gene, since its pseudogene has been annotated as a low-copy repeat (LCR) in the human genome [41].

**Table 3:** Complex rearrangements classification according to pattern similarity

Classification	Description	Number of families
Type 1	*IDS-IDSP1* recombination associated with inversion (including REC Type A, B, C by Lualdi’s method 2005)	130*
Type 2	*IDS-IDSP1* recombination associated with conversion and excision (including REC type D by Lualdi’s method 2005)	2
Type 3	*IDS-IDSP1* recombination associated with deletion (in most cases involving exons 4–7)	20
Type 4	*IDS-IDSP1* recombination not further specified	28
Type 5	Abnormal Southern blot pattern not further investigated or gross gene alteration not further specified	41
Type 6	Well-defined complex rearrangement	14

(* in type 1 rearrangements 2 pts carrying an *IDSP1* duplication are included)

Type 5 and type 6 groups include rearrangements other than the recombinational type: 41 patients with complex rearrangements likely not caused by recombinational mechanisms and not further specified, and only 14 patients in which the rearrangement was well-characterized (at exon or at nucleotide level).

Thus, given the data reported above, if we consider all types of variants, including point variants, large deletions/duplications and complex rearrangements, the most reported variant is the recombination between *IDS* gene and its pseudogene *IDSP1* associated with inversion, with 130 patients carrying this rearrangement. Since this could not be considered a unique variant, as most cases were not characterized at single nucleotide level, the reported ethnicities for this variant were separately described in Table 4.

**Table 4:** Alleles carrying the *IDS-IDSP1* recombination combined with inversion divided by reported ethnicity/country. Only countries/ethnicities with ≥ 3 detected alleles are listed. The other countries/ethnicities are included in the category “*all other countries”*

Alleles by country/ethnicity	Number detected	Percentage of that allele’s total	Percentage of all detected alleles
**Total**	**130**	**100,0**	**4.6**
Chinese	31	23,8	1,1
Russian	14	10,8	0,5
Brazilian	13	10,0	0,5
Japanese	9	6,9	0,3
Italian	7	5,4	0,2
Polish	4	3,1	0,1
Argentinian	3	2,3	0,1
Mexican	3	2,3	0,1
*all other countries*	9	6,9	0,3
*not reported*	37	28,5	1,3

### Geographic distribution of *IDS* variants

Patients’ geographical information was available for 2231 individuals (78.8%), with subjects originating from all continents. Genetic heterogeneity was apparent among all populations. However, if we consider the ratio between the number of unique variants reported in a specific population and the total number of alleles described for the same population, individuals from Mexico, India, Korea and Argentina were the most heterogeneous, whereas those from Russia and Brazil were the least heterogeneous. China and Russia were the most highly represented, contributing 16.3% and 10.2% of all alleles, respectively, followed by Brazil (8.4%), Japan (6.8%) and India (5.8%) (Table 5).

**Table 5:** Most common variants for the 5 most frequent nationalities/ethnic backgrounds reported. For each country, only variants with ≥ 5 detected alleles are listed. The other variants are included in the category “all other variants”

Alleles by country/ethnicity	Number detected	Percentage of that country total	Percentage of all detected alleles
**Chinese**	**455**	**100,0**	**16,2**
*IDS-IDS2* recombination combined with inversion	27	5,9	1,0
c.1402 C > T [p.(Arg468Trp)]	18	4,0	0,6
c.1403G > A [p.(Arg468Gln)]	14	3,1	0,5
c.1122 C > T [p.Glu375_Gly394del]	13	2,9	0,5
complete *IDS* deletion	12	2,6	0,4
c.998 C > T [p.(Ser333Leu)]	12	2,6	0,4
c.1327 C > T [p.(Arg443*)]	11	2,4	0,4
c.262 C > T [p.(Arg88Cys)]	10	2,2	0,4
c.514 C > T [p.(Arg172*)]	8	1,8	0,3
exon 9 deletion	6	1,3	0,2
c.257 C > T [p.(Pro86Leu)]	6	1,3	0,2
*IDS-IDS2* recombination combined with exon 4–7 deletion	5	1,1	0,2
c.1016T > C [p.(Leu339Pro)]	5	1,1	0,2
c.596_599del [p.(Lys199Argfs*13)]	5	1,1	0,2
c.263G > A [p.(Arg88His)]	5	1,1	0,2
c.253G > A [p.(Ala85Thr)]	5	1,1	0,2
*not found/not reported*	6	1,3	0,2
*all other variants*	287	63,1	10,2
**Russian**	**284**	**100**,**0**	**10**,**1**
c.1122 C > T [p.Glu375_Gly394del]	16	5,6	0,6
*IDS-IDS2* recombination combined with inversion	13	4,6	0,5
c.1403G > A [p.(Arg468Gln)]	11	3,9	0,4
c.263G > A [p.(Arg88His)]	9	3,2	0,3
complete *IDS* deletion	9	3,2	0,3
c.514 C > T [p.(Arg172*)]	8	2,8	0,3
c.998 C > T [p.(Ser333Leu)]	8	2,8	0,3
c.262 C > T [p.(Arg88Cys)]	6	2,1	0,2
c.596_599del [p.(Lys199Argfs*13)]	6	2,1	0,2
c.253G > A [p.(Ala85Thr)]	5	1,8	0,2
c.1402 C > T [p.(Arg468Trp)]	5	1,8	0,2
c.1445T > G [p.(Leu482*)]	5	1,8	0,2
*not found/not reported*	9	3,2	0,3
*all other variants*	174	61,3	6,2
**Brazilian**	**235**	**100**,**0**	**8**,**4**
c.230 C > A [p.(Ala77Asp)]	21	8,9	0,7
c.1122 C > T [p.Glu375_Gly394del]	14	6,0	0,5
*IDS-IDS2* recombination combined with inversion	13	5,5	0,5
c.998 C > T [p.(Ser333Leu)]	11	4,7	0,4
c.1327 C > T [p.(Arg443*)]	10	4,3	0,4
c.1402 C > T [p.(Arg468Trp)]	9	3,8	0,3
c.1400 C > T [p.(Pro467Leu)]	7	3,0	0,2
c.262 C > T [p.(Arg88Cys)]	6	2,6	0,2
c.1403G > A [p.(Arg468Gln)]	5	2,1	0,2
c.253G > A [p.(Ala85Thr)]	5	2,1	0,2
*not found/not reported*	8	3,4	0,3
*all other variants*	126	53,6	4,5
**Japanese**	**190**	**100**,**0**	**6**,**8**
*IDS-IDS2* recombination combined to inversion	8	4,2	0,3
Gross gene alteration (not further specified)	8	4,2	0,3
Rearrangement (not further specified)	8	4,2	0,3
Rearrangement (Southern blot analysis)	8	4,2	0,3
c.1403G > A [p.(Arg468Gln)]	5	2,6	0,2
*all other variants*	114	60,0	4,1
*not found/not reported*	39	20,5	1,4
**Indian**	**162**	**100**,**0**	**5**,**8**
c.263G > A [p.(Arg88His)]	11	6,8	0,4
c.1122 C > T [p.Glu375_Gly394del]	10	6,2	0,4
c.998 C > T [p.(Ser333Leu)]	6	3,7	0,2
c.1402 C > T [p.(Arg468Trp)]	6	3,7	0,2
Complex rearrangement (by Lualdi’s method)(not further specified)	6	3,7	0,2
c.253G > A [p.(Ala85Thr)]	5	3,1	0,2
*all other variants*	118	72,8	4,2

### Hunter disease in females

As MPS II is an X-linked inherited disease, it affects principally male children while only 24 cases of female patients (Supplementary File - Table S2) have been so far described in literature. Due to the clinical severity of most patients, coupled with the precocious death, male patients generally do not have children. Therefore, Hunter females very rarely originate from the combination of two mutated alleles, each inherited from one of the 2 parents. They mostly are the result of two different events: the presence of a pathological variant (*de novo* or mother-inherited), and the selective inactivation of the X chromosome (skewed X-inactivation) inherited by the father, thus leading to the expression of the mutated allele in a heterozygous background. Among the 24 cases of Hunter females here reported, in only one case (subject 7) the same missense variant was inherited from both parents, of French Gipsy origin [42], thus being so far the only reported case of homozygous MPS II patient. As for the other 23 cases described, subjects are all carriers of only one mutated allele. Except in one case (subject 20), where the girl was symptomatic due to the presence of only one X chromosome, being a Turner patient [43], all of the other cases likely resulted symptomatic from an imbalanced X-chromosome inactivation. Overall, in the 24 female patients, 20 different variants were identified: 7 missense, 1 nonsense affecting 2 subjects, 3 frameshift, 3 events of recombination between the *IDS* gene and the *IDSP1* pseudogene, 4 large deletions and 2 balanced reciprocal translocations. Eleven patients had inherited the variant, 8 of them represented *de novo* cases, for 2 cases inheritance was uncertain; for the 3 remaining subjects, inheritance from the parents was not assessed.

According to Cook (2019) [44], interchromosomal recombinational events between X chromosome and an autosome may somehow unbalance skewed inactivation, favoring the expression of the recombined chromosomes, thus guaranteeing a balanced situation. Consequently, if any of the genes involved in the recombination event carries a pathogenic variant, the female subject will show the related X-linked disease. In the cohort of females reported in Table S2, this is likely the case of subject 1, carrying a balanced reciprocal translocation between chromosome X and 5, and of subject 18, carrying a *de novo* balanced reciprocal X;9 translocation.

### Genotype-phenotype correlation and in vitro functional studies

Given its X-linked inheritance, genotype-phenotype correlation in MPS II could be considered quite easy as only a single allele has to be correlated to the reported phenotype. However, from a genetic point of view, MPS II is also a very heterogeneous disorder, where only 2.7% of unique variants were reported in a range of frequency from 8 to 118 families (data reported above), and more than 50% of the variants were described in only 1 family. This great heterogeneity makes genotype-phenotype correlation very challenging and in most cases merely unfeasible.

**Fig. 6:**
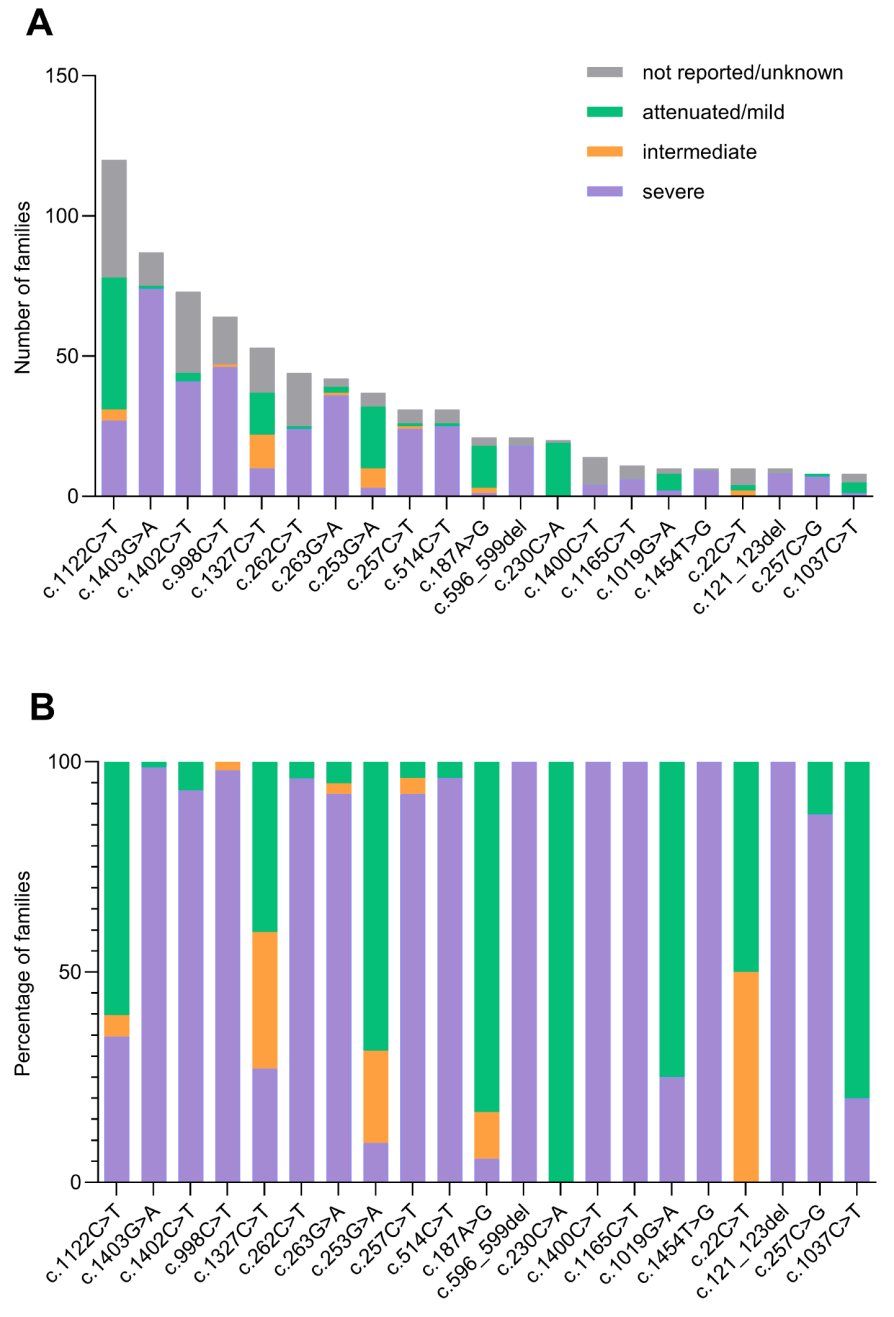
Genotype/phenotype correlation for point variants with frequency above or equal to 8

Moreover, the criteria used to define the clinical phenotypes in literature are not homogeneous and, in most cases, are not reported, especially in the oldest publications. For the 21 unique point variants showing a frequency equal or above 8 families, we collected and analyzed the reported phenotype (Fig. 6a). We evidenced that for 12 variants the severe phenotype was the predominant category. These variants include 8 missense, 2 nonsense, 1 frameshift and 1 inframe deletion. Notably, among the missense variants, c.262C > T and c.263G > A affect the Arg88 residue, located in the catalytic core of the protein [20]. For 6 variants (5 missense and 1 synonymous) the mild phenotype was more frequent than the other categories. Finally, for 3 variants (2 nonsense and 1 missense), the most frequent category was the ‘’unknown/unreported’’ phenotype.

Figure 6b reports these 21 variants analyzed taking into consideration only cases associated with a clinical phenotype (73.9% of families), thus excluding the not reported/unknown phenotypes.

Five variants are associated with severe/neuronopathic phenotypes in all reported cases, 8 variants in ≥ 80% of the cases. Only one variant is associated with attenuated phenotypes in all reported cases and 2 variants in ≥ 80% of the cases. The remaining 5 variants are described in literature with different proportions of discordant phenotypes: severe, intermediate and attenuated.

Overall, families reported with a severe clinical phenotype were 68.3%, the two-thirds of all reported phenotypes, as commonly described for the disease [3, 45].

On the other side, all families reported with the variant c.230 C > A (19 families) present an attenuated phenotype, as well as four out of 5 families carrying the variant c.1037 C > T and 15 out of the 18 cases reported with the variant c.187 A > G; on the whole 38 cases. The remaining five variants (c.1122 C > T, c.1327 C > T, c.253G > A, c.1019G > A, c.22 C > T) are described in literature with different proportions of discordant phenotypes: severe, intermediate and attenuated (Fig. 6b).

However, these analyses should be considered carefully, as for most of the reported phenotype, the criteria used for its definition are not known, especially in the oldest publications.

For 13 of these variants, data from in vitro expression analyses were also available and, in most cases, they were consistent with the reported phenotype. In vitro protein expression and immunoblotting analyses were conducted in several cell types, mainly in COS-1, COS-7, human fibroblasts, CHO, HEK293 cells. Data related to the most frequently reported among them are described below.

The most frequent point variant c.1122C > T, is a synonymous variant in terms of amino acid coding, which however activates a 5’ cryptic splice site inside exon 8, causing the generation of a shorter transcript, lacking the last 60 bp of the exon [45]. Patients’ phenotype is known for 78 out of 118 patients, while for 40 of them phenotype was not described in the original paper. We choose to include in the “unknown phenotypes” also 4 patients whose severity had been based on cardio-pulmonary function, which is not a common criterion to discriminate between attenuated and severe patients, being most, if not all of them, cardio-pulmonary affected. Forty-seven of the 78 patients for whom a clinical phenotype was reported (60.3%) showed an attenuated/mild phenotype. According to Matos et al. (2015), this is due to the presence of some residual IDS activity since together with the mutant splicing form also the correct form is commonly detected in the patients, as previously reported by the same authors [45, 46].

Variant c.1403G > A, [p.(Arg468Gln)] described so far in 87 families, was also analyzed in vitro where IDS enzyme activity, evaluated both in COS cells [47] and in patient’s fibroblasts following transient transfection [48], was detected very low. Immunoblotting analysis, conducted in 1995 by Sukegawa et al., and more recently by Charoenwattanasatien et al., in 2012, also revealed an altered processing of the IDS protein leading to a defective cleavage to the mature form [48, 49]. Seventy-five out of the 87 families carrying this variant were reported with a phenotype, 74 of which (99.7%) associated with a severe clinical form.

Extremely low to absent enzyme activities were shown in vitro following expression of the variants c.1402 C > T [p.(Arg468Trp)], c.1327 C > T [p.(Arg443*)], c.262 C > T [p.(Arg88Cys)], c.253G > A [p.(Ala85Thr)], c.257 C > T [p.(Pro86Leu)] and others. In some of them, in vitro studies also highlighted an altered post-translational processing of the protein. For 2 of these variants, c.1327 C > T and c.253G > A, a discordant phenotype (from attenuated to severe) was reported, while patients carrying the other 3 variants commonly presented with a severe clinical form.

**Fig. 7:**
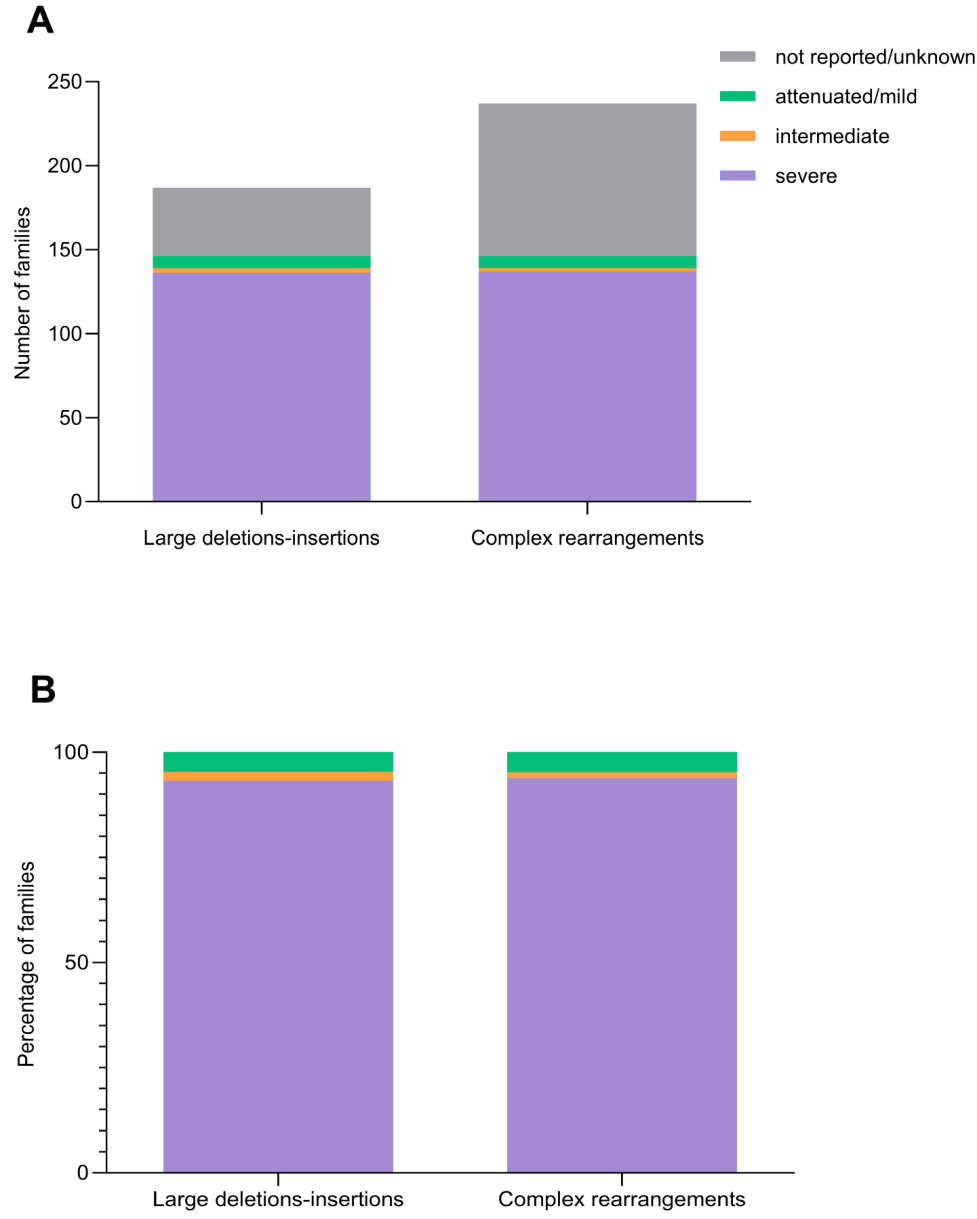
Genotype/phenotype correlation for large deletions/duplications and complex rearrangements

No in vitro expression data was instead available for eight variants shown in Fig. 7. However, for most of them a clear correlation with the phenotype could be observed. The severe phenotype correlated in 100% of the identified cases with the variants c.596_599del and c.1165 C > T, and in most cases carrying the variants c.998 C > T and c.514 C > T. An attenuated phenotype correlated in all the cases carrying the c.230 C > A variant, and in most cases reported with the variants c.187 A > G and c.1037 C > T.

As regarding large deletions/duplications and complex rearrangements, the genotype-phenotype correlation evidenced, as expected, that most patients carrying these types of variants shows a severe phenotype. More precisely, 72.7% of patients carrying large deletions/duplications show a severe phenotype, 3.7% a mild phenotype, 1.6% an intermediate and for 21.9% the phenotype is unknown or is not reported. Similarly, for 57.8% of patients carrying complex rearrangements a severe phenotype is reported, for 3% a mild phenotype, for 0.8% intermediate and for 38.4% is not reported or is unknown (Fig. 7a). Furthermore, if we take into consideration for these two types of variants only patients for whom a clinical phenotype was reported, who are 78.1% for the large deletions/duplications, and 61.6%, for the complex rearrangements, 93.2% and 93.8% of these cases present with a severe/neuronopathic phenotype, respectively (Fig. 7b).

### ACMG classification of *IDS* variants

The ACMG/AMP classification of the 779 *IDS* point variants collected in this study is reported in the Supplementary Table S3. It indicated that for most variants, enough evidence was available to classify them based on their pathogenicity. Indeed, most variants were classified as “pathogenic” (490; 62.9%) and “likely pathogenic” (276; 35.4%), Only, 13 point variants (1.7%) were classified as variants of “uncertain significance.” 768 classified variants and their associated pathogenic evidence were submitted to ClinVar, where they can be retrieved by the following accession numbers: SCV005088913-SCV005089669 and SCV000929879.1-SCV000929889.1. Ten variants were not submitted given their ambiguous annotations which were not accepted by ClinVar and one variant because the URL reference citation was not allowed.

### Genotype and response to ERT

The response to ERT and more specifically the variation between patients in each efficacy outcome, may reflect differences in the age at start of therapy as well as other factors, like the influence of patient’s genotype [50]. Only a few studies evaluated the potential correlation between ERT efficacy and MPS II patients’ genotype. Barbier et al., studied 36 attenuated patients above 5 years of age and found that subjects carrying nonsense or frameshift variants were likely more prone to develop antibodies, infusion-related reactions, and to experience a limited urinary GAG response than those with missense variants [51]. An extension of this study to 27 severe patients and to a higher number of efficacy outcomes [52] evidenced after one year of treatment that patients with complete deletions/large rearrangements (CD/LR) and with frameshift/splice site variants (FS/SSM) had a lower decrease in liver size than those carrying missense variants (MS). Moreover, the average spleen volume was similar in the CD/LR genotype and in the MS genotype groups, but it was significantly higher in the FS/SSM with respect to the other groups. As regarding urinary GAG levels, also in this second study patients with CD/LR evidenced following ERT a less pronounced reduction than patients with MS. In addition, patients with the CD/LR genotype were more likely to develop antibodies to idursulfase than patients with the MS genotype, while the FS/SSM group fell between the CD/LR and the MS groups [52]. Specifically concerning the antibodies, these studies may suggest a limited production of antibodies where the protein, although altered and non-functional, is produced and therefore recognized as self, as in the patients carrying a missense variant. Instead, the total absence of the protein in subjects carrying severe gene alterations, as complete deletions or large rearrangements or frame-shift variants, not allowing the production of the protein, may cause an elevated immune-response against the recombinant IDS, being the protein completely unknown to the immune system, thus representing a non self antigen [51].

However, further studies on larger cohorts of patients are needed to confirm the potential correlations suggested by these studies.

### Open issues

Since the characterization of the region distal to *IDS* gene and the discovering of its pseudogene *IDSP1*, many rearrangements due to intrachromosomal recombinational events between the homologous regions of *IDS* and *IDSP1* have been reported. However, the relative high number of patients with no variants detected (241; 8.6%), reported in this study, as well as in our direct experience, arise the suspect that some recombinational events are still being missed by the standard molecular analysis. Indeed, being these recombinations balanced rearrangements, they are not detected by simple exons sequencing and they require additional approaches such as the rapid RFLP analysis protocol set by Lualdi et al. [53]. This should be a pivotal aspect to take into consideration when approaching the diagnosis of a Hunter patient. In addition, some undetected variants might be deep intronic variants that would be revealed only through second-level molecular analyses. Finally, the molecular analysis of *IDS* gene should not avoid to consider the promoter region of the gene, where a 128 bp deletion was detected in a few mild patients [54, 55].

One additional problem may be represented by the fact that often variants already published are under-reported in the following publications, being considered of limited or no interests for the readers. Such an approach represents a bias in the overall variant analysis, and it is a common problem of several genetic diseases. It causes an underestimate of the frequency of each variant in the general population, as well as in specific ethnic groups. Variants already described, identified in new subjects/families, should at least be communicated to open databases, with some essential demographic, geographical and clinical information. This would certainly change the overall molecular scenario.

Finally, to reduce the risk of variants misreporting, all *IDS* variants should be annotated according to the most recent HGVS nomenclature (Version 20.05), thus allowing an unambiguous and consistent description. To this aim, several in silico tools are available that may support in variants annotating (i.e. Mutalyzer -Name Checker, Variant Validator).

### Future perspectives

As presented in this review, *IDS* is a gene prone to mutate, with more than half variants described in only one family (‘private mutations’). Furthermore, due to the presence of the pseudogene, sharing long sequences with the gene, which favors recombination events, additional genomic changes arise from this phenomenon, often generating genetic alterations difficult to properly define, due to boundaries undefined at nucleotide level. According to our records, patients carrying variants due to events of intrachromosomal recombination so far described account for 180 subjects. A deeper genomic investigation on these variants, as well as on other complex rearrangements or gross gene alterations, should be performed in the light of the new diagnostic approaches, available and more widely used in the last 10–15 years, as the whole genome or the whole exome sequencing analyses or others, depending on the query to be solved.

Update and correct classification of variants characterizing complex genes as *IDS* results very difficult to perform, but also extremely useful, helping to describe a scenario of the gene complexity and providing an updated summary, to be used for diagnostic purposes and in genetic counseling.

Being Hunter syndrome one of the most common LSDs, accounting in some countries for almost half of all MPS cases [56], the inclusion of the disease within the newborn screenings, followed by a second-tier test for confirmation, would be desirable, allowing an early identification of the patients, who still mostly suffer from delayed diagnosis.

An increased reporting of the identified *IDS* variants in public databases should be encouraged, favoring the exchange of information between different laboratories and reference centres, progressively helping a correct and timely molecular diagnosis, and preventing misdiagnoses.

## Electronic supplementary material

Below is the link to the electronic supplementary material.

Supplementary Material 1: Table S1 Title of data: ACMG/AMP Classification Methods. Description of data: Specification of ACMG/AMP criteria applied to IDS variants classification and/or application of the recommendations of the Sequence Variant Interpretation Working Group (SVI WG). Table S2 Title of data: MPS II Female Patients. Description of data: List of MPS II female patients reported in literature. Table S3 Title of data: *IDS* Unique Point Variants. Description of data: List of *IDS* Unique Point Variants collected from literature (last search June 2023). Table S5 Title of data: References Complex Variants. Description of data: References of the articles reporting MPS II patients carrying complex rearrangements. Table S6 Title of data: References Large Deletions-Insertions Variants. Description of data: References of the articles reporting MPS II patients carrying large deletions/insertions.

## Data Availability

The dataset of IDS variants reported in this article is available in the ClinVar repository (https://www.ncbi.nlm.nih.gov/clinvar/). IDS variants can be retrieved by the following accession numbers SCV005088913-SCV005089669 and SCV000929879.1-SCV000929889.1.
